# The Perceived Effectiveness of Various Forms of Feedback on the Acquisition of Technical Skills by Advanced Learners in Simulation-Based Health Professions Education

**DOI:** 10.7759/cureus.44279

**Published:** 2023-08-28

**Authors:** Julia Micallef, Dale Button, Alvaro Uribe Quevedo, Christopher McClatchey, Lindsey King, Adam Dubrowski

**Affiliations:** 1 Health Sciences, Ontario Tech University, Oshawa, CAN; 2 Paramedicine, Durham College, Oshawa, CAN; 3 Software and Informatics, Ontario Tech University, Oshawa, CAN; 4 Paramedicine, Region of Durham Paramedic Services, Whitby, CAN

**Keywords:** technical skill acquisition, intrinsic feedback, augmented feedback, health professions education, simulation

## Abstract

Simulation-based health professions education (SBHPE) is a valuable approach for healthcare professionals to develop and refine technical skills in a safe environment. Feedback plays a crucial role in the acquisition of these skills, but little research has explored the effectiveness of augmented (knowledge of results (KR) and knowledge of performance (KP) versus intrinsic feedback types for advanced learners. Therefore, this study aimed to determine what type of feedback is perceived to be most effective by advanced learners when acquiring complex technical skills in SBHPE. The study followed the test and evaluated phases of the design-based research (DBR) framework. A total of 23 advanced care paramedics (ACPs) participated in the study and received feedback in the form of KR, KP, and intrinsic feedback while using the intraosseous (IO) access simulator. Participants completed a survey to evaluate their learning experience and rank the perceived effectiveness of each feedback type. The results of this study indicated that KP was perceived as the most effective type of feedback and KR was perceived as the least effective feedback, with intrinsic feedback being in the middle. This work provides insights into the use of augmented and intrinsic feedback for advanced learners in an SBHPE environment, but future work to assess the actual learning effects of these types of feedback is needed.

## Introduction

Simulation-based health professions education (SBHPE) employs simulation experiences to enable healthcare professionals to practice technical skills without risking harm to patients [1]. In health professions education (HPE), technical skills, also known as psychomotor skills, encompass the tasks performed by healthcare providers for patients [2]. SBHPE facilitates the acquisition of these technical skills through a three-stage process that ensures a comprehensive understanding of both cognitive and motor aspects: 1) instruction, 2) practice and guidance, and 3) feedback. The instruction and feedback stages contribute to the development of cognitive elements, while the practice stage allows learners to refine their motor skills [3]. This study specifically focuses on the feedback stage.

In this context, there are two distinct categories of feedback: 1) intrinsic feedback and 2) augmented feedback. Intrinsic feedback refers to the sensory information an individual receives during the performance of a psychomotor skill. It includes proprioceptive, visual, and auditory cues that provide real-time information about the execution of the skill. This internal feedback allows individuals to adjust and refine their movements, leading to skill improvement [4]. Augmented feedback, on the other hand, complements intrinsic feedback by offering additional insights into the movement sequence or outcome from a different perspective [4]. It is also known as extrinsic feedback because it comes from an external source, such as a coach, instructor, educator, or technology, rather than from the individual's senses [4]. Augmented feedback can be further classified into two primary dimensions: knowledge of results (KR) and knowledge of performance (KP). KR informs learners about the outcome or consequences of their performance, while KP provides information about the technique, form, or execution of the movement itself [4-5]. To exemplify these feedback types with a basketball player throwing a free throw, intrinsically, the player will be able to see if the ball went into the net or not. With KR, the basketball player’s coach can provide feedback on the parabola of the ball as a result of the throw, or for KP, the basketball coach would provide feedback regarding the basketball player’s form while making the throw to provide an explanation as to why the ball did or did not go into the net.

The role of feedback in technical skills acquisition is crucial; however, no prior research has explored what type of feedback (augmented vs. intrinsic) is most suitable in the context of advanced learners, learning and/or maintaining complex technical skills in SBHPE. In this exploratory study, we focused on the acquisition and maintenance of intraosseous (IO) access skills by advanced care paramedics (ACPs) using an IO access simulator. However, to date, the nature of KR and KP as they relate to IO access skills has not been well defined. Thus, before assessing the perceived effectiveness of each of these types of feedback, a prior study was conducted to establish consensus among expert paramedic educators regarding the definitions of two types of augmented feedback (KP and KR) in this specific context. This process described in the companion paper yielded an eight-point feedback list, comprising both KP and KR, which served as a guideline for paramedic facilitators when offering feedback to ACPs using the IO access simulator [6]. The KP feedback was highly specific, focusing on the correct execution of each step, while KR feedback merely indicated whether the outcome of the step was accurate or not. Subsequently, the objective of this study was to assess which feedback type (KP, KR, or intrinsic) was deemed most effective by ACPs when acquiring IO access skills using an IO access simulator.

## Materials and methods

Design-based research (DBR) is an educational framework that explains an iterative process focusing on the collaboration of researchers, stakeholders, and end-point users, to generate solutions that can be applied to specific learning contexts [7-9]. DBR contains four iterative phases; 1) design, 2) test, 3) evaluate, and 4) reflect [10]. The design phase involves developing a solution that addresses both the theoretical and practical concerns of a problem [8,11]. The test phase involves implementing the solution in a real-world setting [12]. The evaluation phase evaluates the effectiveness of the solution using evidence from endpoint users’ learning [13-14]. Finally, the reflect phase involves a retrospective analysis of the DBR methodology and methods used in the prior phases [14-15]. 

In the first phase of this work, the design phase utilized design thinking and Delphi methods to determine what KP and KR can be provided to ACPs concerning the IO access simulator, as described in a previous article [6]. The second phase, and the focus of this article, was situated within the test and evaluation phases of DBR. The test phase consisted of ACPs receiving feedback in the form of KP and KR, and then comparing them to their intrinsic feedback, when using the IO access simulator. The evaluation phase consisted of ACPs completing a survey to gather demographic data, evaluate the learning experience (using the IO simulator with each type of feedback), as well as ranking their perceived effectiveness of each type of feedback.

Participants

Ethics was obtained for this work by the Durham College Research Ethics Board (241-2122) and approved by the Region of Durham Paramedic Services Ethics Board. Quality and development facilitators (n=2) with an ACP certification from the region of Durham Paramedic Services who are members of the research team (CM and LK) were asked to act as the instructors to provide feedback to the participants. The ACPs (n=23) were recruited from those attending the Region of Durham Paramedic Services' continuing education sessions which occur biannually to introduce and review patient care standards, equipment, and changes to policies and procedures within the organization. Participation in this study was voluntary and each participant was compensated with a $5 Starbucks gift card and entered into a draw to win an iPad. The only inclusion criterion was that the paramedics had to be either an ACP student or a working ACP as paramedics at this level already know the steps involved in performing an IO access.

Procedure

**Figure 1:**
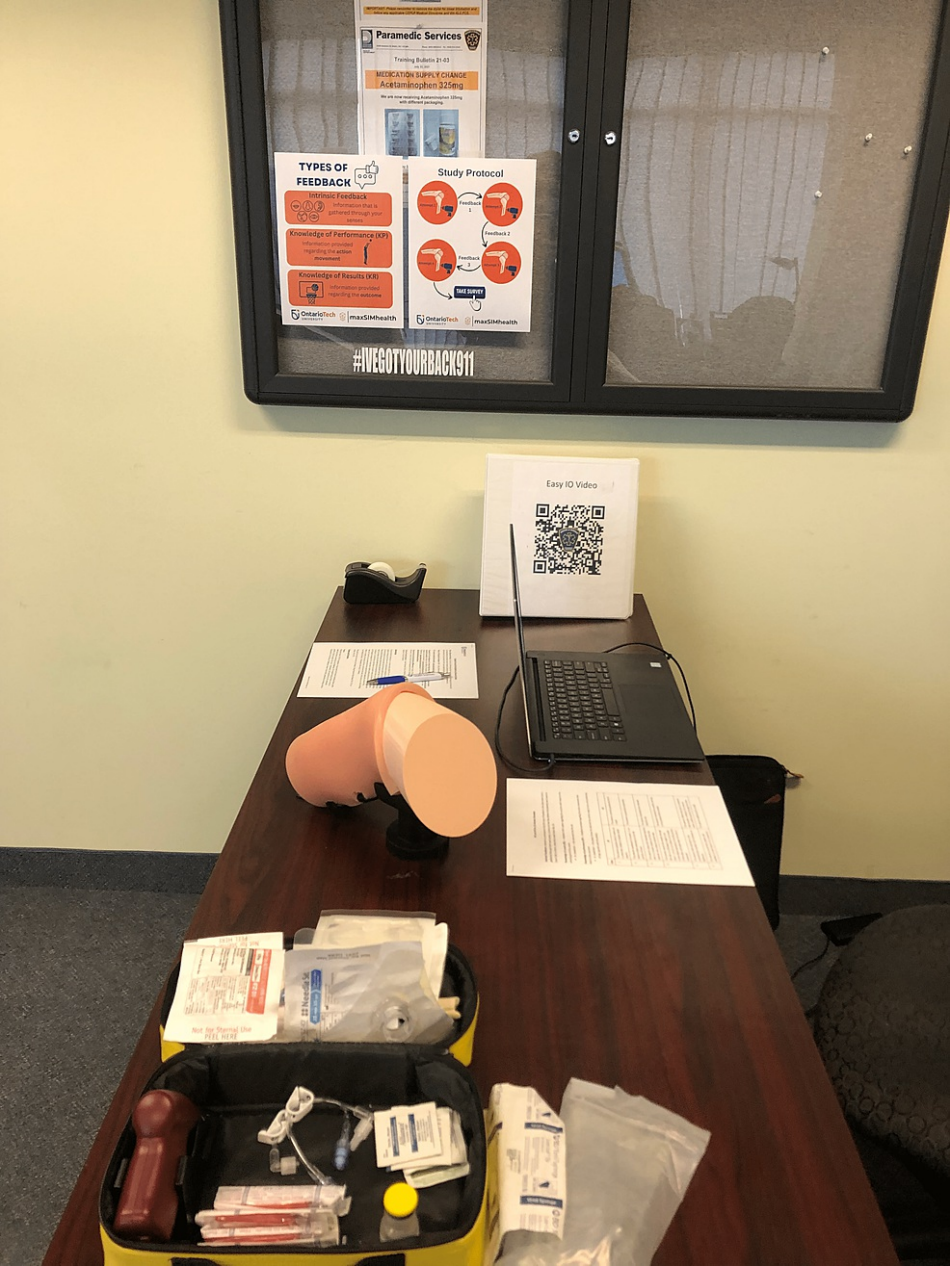
Simulation environment

The simulation environment was set up at the Region of Durham Paramedic Services and included: 1) a poster and cheat sheet explaining the three types of feedback (KP, KR, and intrinsic), 2) a poster outlining the study procedure, 3) an IO access simulator with all of the necessary equipment, and 4) a laptop for the survey (Figure 1). In addition, one instructor (CM or LK) and an additional member of the research team (JM) were present to provide feedback and observe, respectively.

Participants completed the study one at a time. The study lasted a total of approximately 15 minutes, including being briefed by a member of the research team (JM) regarding the different types of feedback they would receive and the study protocol. Each participant provided written consent prior to participation. The protocol required each participant to perform three IO access attempts on the IO access simulator with an instructor (CM or LK) providing one of the three types of feedback in between each attempt, following a guiding script for each type of augmented feedback [16]. The order in which the type of feedback was provided after each attempt was rotated using a Latin square design. The participants were informed by the instructors of what feedback type they would be receiving prior to each attempt, and again right before providing it after each attempt, but they were not aware of the order in which they would receive the feedback at the start of the study. At the end of the practice session, the participants were then asked to complete an online survey (Table 1) to assess the perceived relative effectiveness of each type of feedback. There were three components to the survey as shown in Table 1: 1) demographic data, 2) self-efficacy data regarding the learning experience, and 3) indicating which feedback was most effective and why. The demographic data collected aimed to gather information on how experienced the ACP was in performing an IO access. The self-efficacy component was based on the Michigan Standard Simulation Experience Scale, which is used to gather perspectives on SBHPE environments, to assess whether participants found the learning experience (using the IO access simulator while receiving feedback) helped improve their knowledge, confidence, and ability in performing the IO access procedure [17]. Participants had to rank each question on a five-point Likert scale with one being strongly disagree and five being strongly agree. The final component of the survey required participants to select which feedback was most effective and which was least effective, and then explain their selections in an open-ended question format.

**Table 1:** Survey given to participants to assess the simulation environment and the perceived relative effectiveness of each type of feedback IO, intraosseous

Question #	Question
	DEMOGRAPHIC QUESTIONS
1	How many years have you been in practice as an ACP? Please indicate full-time or part-time.
2	How many IOs have you done in your career?
3	I attempted an IO in the last ...
4	What is your perceived ability to perform IOs?
	SELF-EFFICACY QUESTIONS
5	This learning experience helped improve my KNOWLEDGE on the procedure in scope.
6	This learning experience helped improve my CONFIDENCE in performing the procedure in scope.
7	This learning experience helped improve my ABILITY in performing the procedure in scope.
	FEEDBACK QUESTIONS
8	Which type of feedback was the MOST effective for you?
9	Which type of feedback was the LEAST effective for you?
10	Please explain your reasoning for your response to questions 8 and 9.

In addition, after the data was collected for each of the ACPs, an online survey was given to the two instructors to assess their perspective of the experience by ranking on a five-point Likert scale from one (strongly disagree) to five (strongly agree), to determine which feedback was easiest for them to provide to learners, and to express which feedback they believed was most effective for the learners. The questions are shown in Table 2.

**Table 2:** Survey given to instructors to assess their perceptions of the learning experience

Question #	Question
1	It was easy to follow the script developed from the design thinking and Delphi rounds.
2	The learning experience (using the IO simulator with your feedback) was a beneficial training experience for you.
3	Was it easier to provide participants with KP (knowledge of performance) or KR (knowledge of results)?
4	Which feedback do YOU think the participants liked the most?
5	Please explain your answers for the questions above, and provide any additional comments you would like to add regarding this experience.

Variables of interest and data analysis

The self-efficacy data was considered ordinal; therefore, the median and standard deviations (SD) for each question were calculated. The feedback ranking data was separated into quantitative and qualitative data. The quantitative data from the surveys were analyzed using a chi-square analysis and the qualitative data were thematically analyzed. The instructor data were not analyzed using inferential statistics, as there were only two individuals enrolled in the study. However, the results were utilized to provide some insight into the different perspectives of the learning experience.

## Results

**Figure 2:**
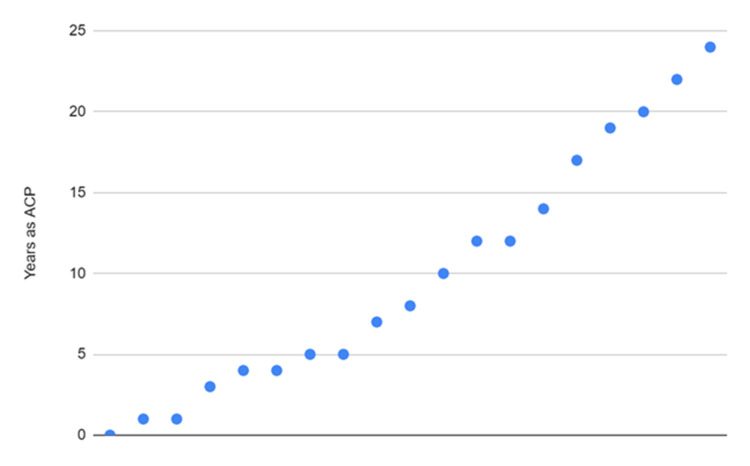
Years as advanced care paramedics as indicated by participants ACP, advanced care paramedic

**Figure 3:**
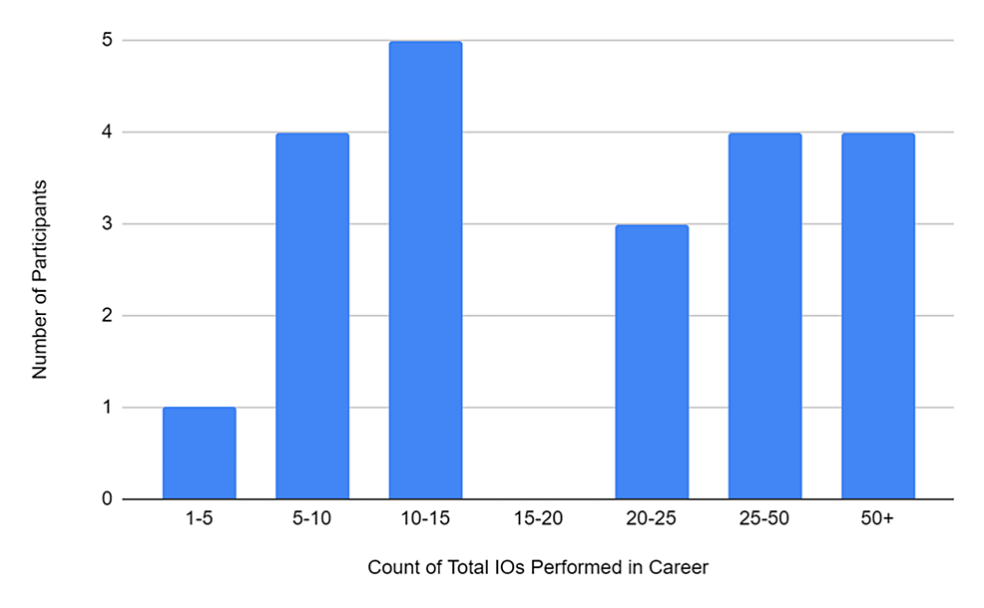
Summary of the participants’ count of estimated total of IO accesses performed in their career IO, intraosseous

**Figure 4:**
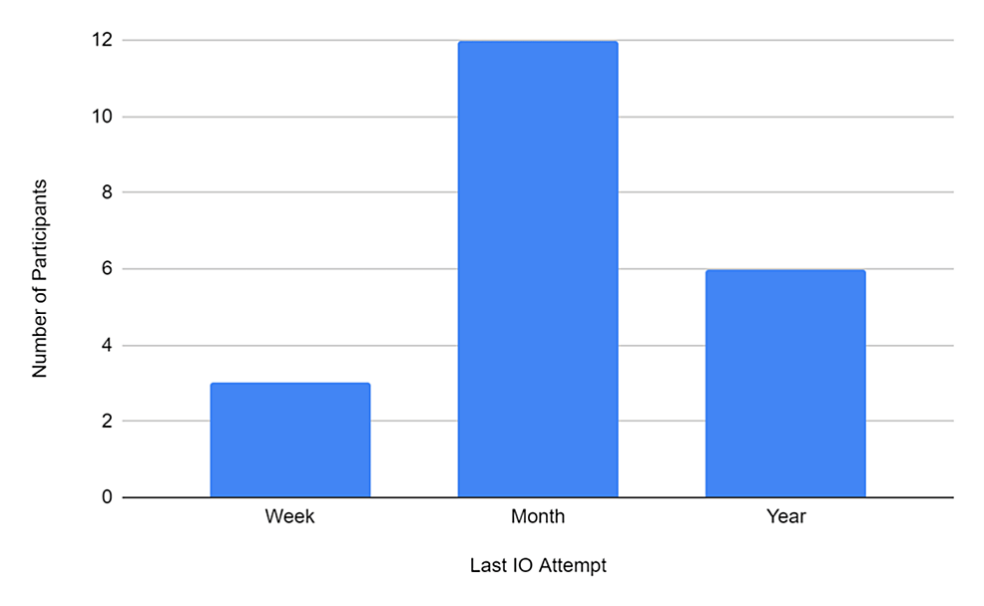
Participants’ last IO attempt before participation in the study IO, intraosseous

**Figure 5:**
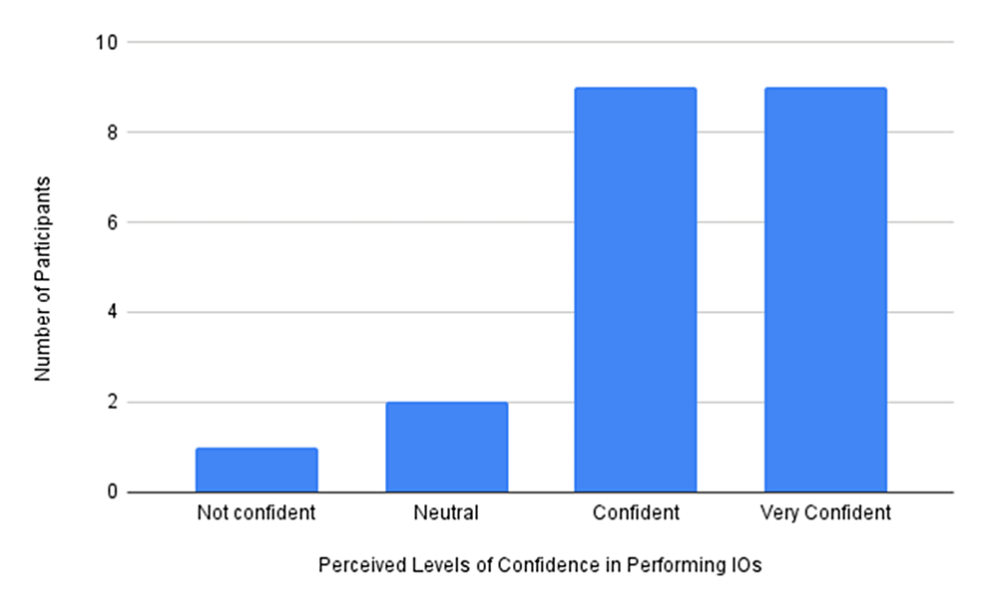
The participants’ perceived level of confidence in performing IOs IO, intraosseous

The demographic data are illustrated in Figures 2-5. Figure 2 shows a plot indicating how many years the participants have been an ACP ranging from 0 years (an ACP student) to 24 years. 

Figure 3 shows how many estimated IOs have been performed by the participants throughout their careers. The most prominent range of total IOs performed was 10-15 with five participants.

Figure 4 shows when the participants last attempted an IO in practice before participating in the study. The most prominent response was that 12 participants selected that they had performed an IO within the last month prior to participation in the study.

Figure 5 shows the participants' perceived confidence in performing IOs. As seen in the figure, 18 participants indicated that they were either confident (n=9) or very confident (n=9) in performing IO access skills. 

Self-efficacy of the simulation environment

The data are presented in Table 3 as frequencies, medians, and standard deviations. Overall, the participants thought that the learning experience (using the IO access simulator with feedback from an instructor) helped improve their knowledge (median=4, SD=0.470), confidence (median=4, SD=0.600), and ability to perform the procedure (median=4, SD=0.733), indicating that the SBHPE environment was beneficial to improving their self-efficacy in performing this procedure.

**Table 3:** Self-efficacy data with outliers as medians

	This learning experience helped improve my KNOWLEDGE on the procedure in scope	This learning experience helped improve my CONFIDENCE in performing the procedure in scope	This learning experience helped improve my ABILITY in performing the procedure in scope
Strongly disagree (1)	0	0	0
Disagree (2)	0	0	1
Neutral (3)	0	2	4
Agree (4)	16	14	14
Strongly agree (5)	7	7	4
Data analysis			
Median	4.000	4.000	4.000
Standard deviation	0.470	0.600	0.733

Perceived effectiveness of feedback data

Quantitative Analysis

The quantitative results shown in Table 4 indicate that KP was perceived as the most effective feedback and KR was perceived as the least effective feedback (p=0.0003).

**Table 4:** Most effective versus least effective feedback and chi-square analysis KP, knowledge of performance; KR, knowledge of results; X^2^, chi-square; DoF, degrees of freedom; p, p-value

Type of feedback	Most effective	Least effective
KP	15	3
KR	2	13
Intrinsic	6	7
Data analysis		
X^2^	16.1436	
DoF	2	
p	0.0003	

Qualitative Analysis

Three main themes emerged from the qualitative analysis, which revolves around the types of feedback received (KP, KR, intrinsic), their perceived value or effectiveness, and the role of feedback in learning, adjustment, and improvement. The three themes are 1) the value of KP, 2) the limitations of KR or intrinsic feedback, and 3) the importance of feedback for learning and improvement. These themes with supporting quotes are shown in Table 5. For the first theme (value of KP), several comments highlighted the importance and effectiveness of receiving specific and detailed feedback on the procedure, technique, and steps involved in performing a skill. Participants appreciated the ability to adjust, improve, and learn from their mistakes based on this type of feedback. For the second theme, limitations of KR or intrinsic feedback, some comments expressed less value or effectiveness attributed to KR or intrinsic feedback. KR, which provides information on whether the skill was performed correctly or incorrectly, is seen as less informative and lacking in instructional guidance. Intrinsic feedback, based on personal assessment or feeling, is considered subjective and may not necessarily lead to recognizing inefficiencies or areas for improvement. Finally, the third theme, the importance of feedback for learning and improvement, was highlighted by many participants emphasizing the significance of feedback in the learning process and improving skills. Feedback, particularly KP, is viewed as valuable for adjusting and refining techniques, understanding specific steps, and enhancing overall performance. The ability to learn from feedback and make corrections for future attempts is seen as crucial for skill development.

**Table 5:** Themes and supporting quotes from the open-ended questions in the survey

Themes	Supporting Quotes
Value of KP	“Knowledge of Performance was most effective because it outlined the specifics that needed to be changed or repeated to correctly perform the given skill” (participant #2), “[Knowledge of] Performance was more valuable as I was able to correct my initial attempt and remark my needle positioning” (participant #11), “Knowledge of Performance gave me information on the actual procedure and where I went right/wrong” (participant #4), “The most effective feedback for me was knowledge of performance. I enjoyed having the very specific guidelines given to me so that I could assess how I was doing directly after performing the skill. I was able to create a checklist in my head of what was good and what needed improvement” (participant #21),
Limitations of KR or intrinsic feedback	“I believe that the knowledge of results was least informative as it just tells me whether I did it right or wrong” (participant #4), “The least effective was the knowledge of results as it seemed very 'cold' and 'clinical' and lacked humanity” (participant #17), “Intrinsic feedback was least valuable because the skill may have been performed incorrectly even if based off of my assessment it appeared to be done right” (participant #2), “Intrinsic feedback didn't provide the reassurance and confirmation that would increase my confidence in a skill” (participant #23).
Importance of feedback for learning and improvement	“I appreciate hearing feedback; you can learn from feedback, improve on mistakes” (participant #20), “Knowing why a skill was done right or wrong helps to better correct wrong steps” (participant #18), “With Knowledge of Performance, you can learn from your feedback and improve on skills in the future” (participant #12), “I found that the location [of IO needle insertion] was much better positioned when I did in fact listen to the feedback about needing more external rotation” (participant #14).

Apart from these main themes, some nuanced and interesting topics emerged from the open-ended question. First is that there are individual learning preferences that affect which feedback is most effective for the participants. Several participants mentioned their preference for detailed feedback and a step-by-step breakdown of the skill, as indicated by the first theme in Table 5. On the other hand, some participants valued a more general overview and reinforcement of their existing knowledge, with one participant noting that, “knowledge of results helped to reinforce my existing knowledge without overwhelming with other information and details” (participant #22). Another topic that arose with some of the more experienced learners (17+ years of experience) was that they provided some insight into why intrinsic feedback may be favorably compared to augmented feedback. One participant indicated, “I think my intrinsic feedback was more valuable than KR due to the fact I have done it multiple times so using a training adjunct I can feel to landmark, feel the pop of the IO gives me more value than just being told I did it correctly” (participant #1). Similarly, another participant noted, “I think KP is very important for a new learner but for someone who does IO regularly or an experienced provider they might be comfortable with the steps but need that feedback of the results” (participant #10). Finally, an interesting point emerged with how the feedback received when using the IO access simulator would differ when performing this skill on an actual patient. The participant first explained that KP was more effective for them when using the IO access simulator, then went on to say that, “...although I am very intrinsic in terms of how my practice is, and on real patients, it is easier to tell what is working and what is not working, however on the training tool there was little ability to see if the line flushed well, or medication was able to be administered” (participant #21). 

Instructor perspectives on learning experience

The responses from both instructors were identical to each other in that they both strongly agreed that it was easy to follow the feedback script provided to them and that the learning experience was a beneficial training experience. Additionally, both instructors found that it was easier to provide KP in comparison to KR and that they believed that KP was liked by participants the most in comparison to KR and intrinsic feedback. When providing reasoning for this, one instructor indicated, “I truly think students want detailed feedback, especially this generation. But having that intrinsic feedback where they get to see their 'poor placement' is beneficial to their learning and practice” (instructor X). On a similar note, the other instructor commented, “I suspect most participants would prefer KP or intrinsic feedback. KP would likely be most beneficial for the new learner, with intrinsic feedback being appreciated by the experienced learner” (instructor Y). When discussing the overall experience, it was suggested, “to provide a bit more 'training', just to ensure more consistency between myself and the other administrators of the feedback” (instructor Y).

## Discussion

The overarching aim of this work was to answer the question of what type of feedback (KR, KP, intrinsic) is perceived to be most effective when acquiring complex technical skills by advanced learners in the context of SBHPE of learning IO access skills by ACPs. To accomplish this, we first had to operationally define two types of augmented feedback (KP and KR) in this context which was described in the companion paper [6]. In this article, we describe the second phase of this work where ACPs from the Region of Durham Paramedic Services used an IO access simulator and received all three forms of feedback (KP, KR, and intrinsic) from paramedic educators. The results from this study indicated that KP was perceived to be the most effective, then intrinsic, and then KR as the least effective.

The results of this work can be interpreted and provide implications methodologically, theoretically, and practically. The methodological contributions of this work revolve around utilizing the DBR approach to develop feedback in SBHPE. DBR was originally introduced by Ann Brown in the early 1990s as a collaborative method for creating interventions in educational settings [13]. While traditionally used in conventional learning environments (such as in classrooms to develop curricula), DBR can also be applied to experiential learning, just as it was in this work for an SBHPE context. Similarly, Schmitz et al. used DBR to develop and assess a cardiopulmonary resuscitation training approach, called *HeartRun*, for school children [18]. Similar to Schmitz et al., this work also used DBR to both develop a new training approach (feedback to suit the specific SBHPE context of ACPs learning IO access skills using a procedural simulator) and assess the perceived learning using the said approach [18]. While Schmitz and colleagues collected their quantitative data via self-assessed learning outcome questionnaires and qualitative data via interviews, this work collected both qualitative and quantitative data in the form of a survey that had both rankings on a five-point Likert scale and open-ended questions. 

Theoretically, this work advances the field of SBHPE and feedback, by providing early evidence about what type of feedback to use when teaching advanced learners technical skills in an SBHPE environment. A key finding from this research was that intrinsic feedback was perceived to be less effective than KP (perceived to be most effective) but more effective than KR (perceived to be least effective). Intrinsic feedback does not involve formal evaluation but requires the learner to be able to identify their errors and learn from them [4]. In this work, participants were allowed to take apart the IO access simulator and get a better look at their landmarking to be able to identify where they went wrong. One possible explanation for this finding is that the ACPs may have the experience and advanced knowledge to know what they did wrong between attempts and can correct themselves. There are two bodies of evidence that can help with the interpretation of these results: KP vs. KR, and self-regulation in SBHPE. When comparing KP and KR, previous research shows that for skills that require technique and precision, KP tends to be more effective than KR [19]. This is in line with the findings of this research concerning KP being ranked as most effective and KR being ranked as least effective because IO access skills require technique and precision to perform successfully [19]. However, the systematic review by Oppici et al. indicated that these findings can only be generalized to novice learners, as well as the findings concerning studies that assess these types of feedback in learning athletic and exercise tasks, as with the majority of the research done in this field [19]. Therefore, the results of this work support and extend these effects to highly skilled learners in a SBHPE environment, who undergo maintenance training on complex psychomotor tasks. Self-regulation and intrinsic feedback play crucial roles in the acquisition and refinement of psychomotor skills, and the interplay of these two can provide some insight as to why intrinsic feedback was perceived to be more effective than KR. Self-regulation refers to an individual's ability to monitor, control, and adjust their behavior, thoughts, and emotions during the learning process [20]. In the context of psychomotor skills acquisition, self-regulation involves the ability to regulate one's movements, actions, and strategies to improve performance [8]. Intrinsic feedback is the sensory information received by an individual’s senses during the performance of a psychomotor skill, which is why we allowed participants to not only reflect on how the procedure felt but let them fully examine the IO access simulator to see where they potentially went wrong or to confirm they did it right [4]. Intrinsic feedback provides real-time information about the quality, accuracy, and effectiveness of the skill execution [4]. Research has highlighted that while intrinsic feedback is crucial, external feedback from coaches, instructors, or peers also plays a role in self-regulation by providing additional information, guidance, and perspectives that complement intrinsic feedback [4,8,20-21]. As seen in the results of this work, despite the participants all being considered advanced learners who can effectively utilize their intrinsic feedback and self-regulate their errors based on their experiences, the feedback perceived to be most effective as a type of augmented feedback (KP, specifically) provided by the instructors. Effective self-regulation involves integrating external feedback with intrinsic feedback for comprehensive skill development. Within the field of SBHPE, providing feedback to learners is a crucial aspect of simulation education, as it generates enduring learning outcomes, enables students to gain an understanding of their performance, and helps mitigate the decline in knowledge retention over time [22-23]. Feedback in simulation usually comes in the form of checklists such as global rating scales as well as provided verbally free form [22,24]. However, no research looks into how KP, KR, and intrinsic feedback can be utilized in SBHPE environments. Therefore, there is a significant gap that this work begins to close through the comparison of augmented forms of feedback (KP and KR) with intrinsic feedback.

Practically, this work highlights the importance of the need to provide training regarding educational concepts such as KP, KR, and intrinsic feedback for this to be implemented in the curriculum and increase the uptake of educators. Within this work, it was noted in the instructor survey that despite us providing some base knowledge on these feedback types, more training was needed for how to provide the feedback. This is in line with the Consolidated Framework for Implementation Research, an implementation framework, which has the domain of providing access to knowledge and information through training so that the innovation can be properly implemented and delivered [25]. Through doing this, educational materials can be created for educators that can highlight the main concepts of how to teach. Another practical implication of this work is how simulations can be developed to inform learners about outcomes and be able to use their existing knowledge of intrinsic feedback to self-regulate. Having the IO access simulator developed in a manner that allowed the participants the freedom to take it apart and be able to reflect and adjust their performance was shown to be a helpful component of the learning process as indicated by the participants. Finally, while advanced learners can use their experiences to reflect and adjust their performance using intrinsic feedback, as per the results of this work, receiving augmented feedback in the form of KP is still important, even for advanced learners. While many of the participants, as well as the instructors, indicated that the more experienced ACPs would appreciate relying on their intrinsic feedback as this is what they utilize in the field, using a simulator is different from performing this skill on a patient and involves a learning curve for even the most experienced participants. Therefore, the augmented feedback helped with this learning curve and possibly provides some rationale as to why KP in an SBHPE environment was still the most preferred compared to intrinsic feedback.

The main limitation of this work is that only the perceived effectiveness of the different forms of feedback was assessed without assessing actual effects on learning, which is usually done through retention or transfer tests. However, due to time constraints with the participants, we were unable to do this. Our future work will employ an experimental design that would allow us to assess this. Another potential limitation is that we had to rely on the participants' understanding of the different types of feedback. For many, this study was the first time they heard of these types of feedback. To mitigate this, we provided an oral overview of the feedback types prior to participation, with a visual poster to reference throughout the study, as well as a cheat sheet with the definitions and the feedback given to them while filling out the survey.

## Conclusions

This study aimed to assess the perceived effectiveness of different types of feedback (KP, KR, and intrinsic) for ACPs acquiring IO access skills using an IO access simulator. The study followed the test and evaluation phases of the DBR framework where instructors provided augmented feedback to the participants while they used the IO access simulator and compared it to their intrinsic feedback. KP was found to be most effective followed by intrinsic, and KR was perceived as least effective. Overall, this study contributes to the existing knowledge in the field of feedback by exploring the perceived effectiveness of both augmented and intrinsic feedback in an SBHPE context. Further research to compare the actual learning effects of these types of feedback will provide a comprehensive understanding of which feedback type for advanced learners in SBHPE is best, ultimately enhancing the training and development of healthcare professionals.
